# Diversification of the phaseoloid legumes: effects of climate change, range expansion and habit shift

**DOI:** 10.3389/fpls.2013.00386

**Published:** 2013-10-09

**Authors:** Honglei Li, Wei Wang, Li Lin, Xiangyun Zhu, Jianhua Li, Xinyu Zhu, Zhiduan Chen

**Affiliations:** ^1^State Key Laboratory of Systematic and Evolutionary Botany, Institute of Botany, Chinese Academy of SciencesBeijing, China; ^2^Graduate School of Chinese Academy of SciencesBeijing, China; ^3^Biology Department, Hope CollegeHolland, MI, USA; ^4^School of Life Sciences, Nantong UniversityNantong, China

**Keywords:** aridification, biogeography, dispersal, diversification rate, habit shift, Leguminosae, molecular dating

## Abstract

Understanding which factors have driven the evolutionary success of a group is a fundamental question in biology. Angiosperms are the most successful group in plants and have radiated and adapted to various habitats. Among angiosperms, legumes are a good example for such successful radiation and adaptation. We here investigated how the interplay of past climate changes, geographical expansion and habit shifts has promoted diversification of the phaseoloid legumes, one of the largest clades in the Leguminosae. Using a comprehensive genus-level phylogeny from three plastid markers, we estimate divergence times, infer habit shifts, test the phylogenetic and temporal diversification heterogeneity, and reconstruct ancestral biogeographical ranges. We found that the phaseoloid lineages underwent twice dramatic accumulation. During the Late Oligocene, at least six woody clades rapidly diverged, perhaps in response to the Late Oligocene warming and aridity, and a result of rapidly exploiting new ecological opportunities in Asia, Africa and Australia. The most speciose lineage is herbaceous and began to rapidly diversify since the Early Miocene, which was likely ascribed to arid climates, along with the expansion of seasonally dry tropical forests in Africa, Asia, and America. The phaseoloid group provides an excellent case supporting the idea that the interplay of ecological opportunities and key innovations drives the evolutionary success.

## Introduction

Species diversity of different lineages inhabiting the Earth is strikingly heterogeneous. The evolutionary success is responsible for those speciose lineages, but discerning what forces have driven the evolutionary success is a major challenge in evolutionary biology (Schluter, 2000). Recently, ecological opportunity and/or key innovation have been considered as a cause of high diversification rates in many groups (e.g., Yoder et al., 2010; Duputié et al., 2011; Claramunt et al., 2012; Erkens et al., 2012). However, little is known about how ecological opportunity and key innovation have interplayed to promote diversification of a group.

The origin and diversification of angiosperms since the Mesozoic are the great events in the plant kingdom. Angiosperms now include about 300,000 species, have taken various ecological habitats on the Earth, and supplied most raw materials for the well-being of human beings. The family Leguminosae is especially outstanding in this term. The phaseoloid legumes represent one of the largest clades in Leguminosae and consist of 114 genera with *ca*. 2000 species (Table S1; Lewis et al., 2005). This clade contains many economically important species, such as soybean (*Glycine max*), common bean (*Phaseolus vulgaris*), cowpea (*Vigna unguiculata*), pigeonpea (*Cajanus cajan*), horse gram (*Macrotyloma uniflorum*), siratro (*Macroptilium atropurpureum*), and coral tree (*Erythrina* spp.) (Bruneau and Doyle, 1990). Molecular phylogenetic studies have contributed greatly to the delimitation of the clade (Lavin et al., 1998; Hu et al., 2000; Lee and Hymowitz, 2001; Doyle et al., 2003; Wojciechowski et al., 2004; Lewis et al., 2005; Stefanović et al., 2009). Now, the phaseoloid legumes contain four subtribes of Phaseoleae, viz. Phaseolinae, Glycininae, Cajaninae, and Kennediinae, and Psoraleeae and Desmodieae (Lewis et al., 2005; Stefanović et al., 2009). The aforementioned phylogenetic analyses of the clade usually sampled less than 40% of the generic diversity in the group; thus, to understand the evolutionary dynamics responsible for its current diversity we need a well-resolved phylogeny of the phaseoloid genera with more comprehensive taxon sampling.

Based on an analysis of *matK* sequences with 13 fossil calibration points, Lavin et al. (2005) suggest that the phaseoloid legumes became differentiated in the Oligocene (24.2–32.1 Ma). Since the Oligocene, global climate has undergone marked changes (Zachos et al., 2001; Wade and Pälike, 2004; Pälike et al., 2006), which may have impacted speciation of many organisms. Egan and Crandall (2008) assume that the recent rapid radiation of Psoraleeae of the phaseoloid legumes may be due to global climate change during the Pleistocene. The phaseoloid legumes possess both woody and herbaceous habits and are primarily distributed in tropical and temperate forests or grasslands (Table S1; Sprent, 2007). The habit shifts have been suggested being responsible for the diversification of some angiosperm lineages (Tiffney and Mazer, 1995; Verdú, 2002; Jabbour and Renner, 2012). Nevertheless, it remains puzzling and unexplored how evolution of habits fostered diversification in the phaseoloid legumes and how ecological forces have been regulating cladogenesis in different geographical areas.

In this study, we first reconstruct a genus-level phylogeny for the phaseoloid legumes using three plastid loci with a more extensive generic sampling than in any previous studies. In the improved phylogenetic framework, we then explore how the interplay of past climate changes, geographical expansion and habit shifts may have triggered diversification of the phaseoloid legumes.

## Materials and methods

### Taxon sampling

We sampled 85 species from 82 of the 115 genera of the phaseoloid legumes. Our worldwide taxon sampling scheme covered all tribes and subtribes of Lewis et al. (2005) and major clades of Stefanović et al. (2009) in the phaseoloid legumes. Our outgroups included thirteen species, representing the other three subtribes of Phaseoleae, Diocleinae (four species), Clitoriinae (two species), and Ophrestiinae (one species), which are excluded from the phaseoloid legumes; Millettieae (five species) and Abreae (one species), following the results of Wojciechowski et al. (2004). Voucher information and GenBank accession numbers are listed in Table S2.

### Laboratory protocols

Three chloroplast markers were used in this study: *rbcL, trnL-F* region (*trnL* intron, and *trnL* [UAA] 3′ exon-*trn*F [GAA] intergenic spacer), and *trnK*/*matK* region (including most of the 3′ *flanking trnK* intron and the entire *matK* gene).

Genomic DNA was isolated from silica-gel-dried materials using a Plant Genomic DNA Kit (Beijing Biomed Co., LTD, BJ, China) or from herbarium samples following a modified CTAB procedure (Doyle and Doyle, 1987). Three DNA regions were amplified with the polymerase chain reaction (PCR). The primers used in this study are listed in Table S3. PCR amplifications were performed using 2 × Taq PCR MasterMix (Beijing Biomed Co., LTD) in 25-μL reactions with the following thermocycler program: 2 min at 95°C for denaturation, then 35 cycles of 30 s at 95°C, 30–60 s at 53–57°C for annealing, 2 min 30 s at 72°C for primer extension, and a 10-min incubation at 72°C following the cycles. The PCR products were purified using a GFX™ PCR DNA and Gel Band Purification Kit (Amersham Pharmacia Biotech, Piscataway, NJ, USA) and then directly sequenced. Sequencing reactions were conducted using an ABI Prism BigDye Terminator Cycle Sequencing Kit (Applied Biosystems, ABI, BJ, China). Sequences were analyzed using ABI 3730 × l DNA Analysis Systems and following the manufacturer's protocols.

### Phylogeny and divergence time estimates

Sequence alignments were done using CLUSTAL X v2.0 (Larkin et al., 2007) and manually adjusted with BioEdit v5.0.9 (Hall, 1999). All alignments are available upon request from the corresponding author. We used the Bayesian relaxed clock methodology as implemented in BEAST v1.7.5 (Drummond et al., 2012) to generate a dated phylogeny of the phaseoloid legumes. The GTR + I + Γ model was selected as the best-fit model for each plastid region determined by ModelTest v3.7 (Posada and Crandall, 1998). Base frequencies were estimated. Clock rate was estimated under an uncorrelated relaxed-clock log-normal (UCLN) model. A Yule speciation model was used as a prior on the tree.

Some fruit and leaf fossils of *Pueraria* of the phaseoloids from the Middle Miocene of middle latitudes in Asia have been described (Wang et al., 2010, and references therein), but we did not use them as calibration points because *Pueraria* is not monophyletic (Lee and Hymowitz, 2001; Stefanović et al., 2009; this study). Employing 13 fossil age constraints imposed on the *matK* phylogeny, Lavin et al. (2005) provide a credible age framework for Leguminosae. Following the results of Lavin et al. (2005), we selected six calibration points: (1) a 45.2 Ma constraint on the root age (node 1); (2) the split between *Platycyamus regnellii* and the phaseoloid legumes (node 2) was set to 39.7 Ma; (3) an age of 27.8 Ma to constrain the crown group age of the phaseoloid legumes (node 3); (4) the crown group age of Desmodieae (node 4) was set to 14.2 Ma; (5) the crown group age of clade VIII (node 5, Figure S1) was set to 19.2 Ma; and (6) an age of 6.3 Ma for the crown group age of tribe Psoraleeae (node 6). A normal distribution was used for all six calibration points. The standard deviation was set to contain the lower and higher boundaries of the 95% highest posterior density values. MCMC searches were run for 100,000,000 generations, sampled every 1000 generations. Tracer v1.5 was used to monitor appropriate burn-in and the adequate effective sample sizes of the posterior distribution (>200). The maximum clade credibility tree was computed by TreeAnnotator v1.7.5 in BEAST software package (Drummond et al., 2012). BEAST analyses were performed in the CIPRES Web Portal 3.1 (Miller et al., 2010).

### Habit evolution

The reconstruction of habit evolution in the phaseoloid legumes were carried out using the parsimony method with Mesquite v2.74 (Maddison and Maddison, 2009). The maximum clade credibility tree obtained from BEAST was used in the analysis. Two habit states were scored, herbaceous (including herbs and herbaceous climbing vines) vs. woody (including trees, woody climbers, and shrubs), based on the literature (Table S2).

### Diversification analyses

To visualize the temporal variation in diversification rates, semilogarithmic lineage-through-time (LTT) plots were constructed in the R package APE v2.5-1 (Paradis et al., 2004). To evaluate 95% credibility interval of the empirical LTT curve, 1000 ultrametric trees randomly sampled from the converged BEAST trees were also used to calculate semilogarithmic LTT plots.

To detect rapid shifts in diversification rates at any specified time, the RC statistic was calculated with the R package GEIGER v1.3-1 (Harmon et al., 2008). Lineages with more or fewer descendents than expected under the constant rate model were hypothesized as a diversification rate shift. Species diversity for the phaseoloid legumes was estimated from the number of species in each genus; missing genera were assigned to corresponding clades based on previous studies (Table S1). Net diversification rates (*r*) for the phaseoloid legumes, nodes R1 and R2 were calculated by using BEAST chronogram under two extremes of the relative extinction rate (ε = 0 and 0.9) following the whole-clade method (Magallón and Sanderson, 2001). Calculations were performed using the GEIGER v1.3-1 (Harmon et al., 2008).

### Biogeographical analyses

To reconstruct the possible ancestral ranges of the phaseoloid legumes, we conducted a Bayes-DIVA analysis (Nylander et al., 2008) using the software package RASP (Yu et al., 2011). Bayes-DIVA method can minimize the phylogenetic uncertainties by utilizing the posterior distribution of trees resulting from a BEAST analysis and generating credibility support values for alternative phylogenetic relationships (Nylander et al., 2008; Yu et al., 2011). We randomly sampled 1000 trees from the BEAST output as a “trees file” and used the maximum clade credibility (MCC) tree as a final representative tree. Biogeographical analyses were conducted on continental spatial scale at generic level, because the aim was to predicate the ancestral areas of nodes deeper down into the tree other than the ancestral areas of individual genera. Six geographic regions were coded: A, Asia; B, Africa; C, Europe; D, Australia; E, South America; F, North America (including Central America and Caribbean). Ancestral areas were reconstructed with the “maxareas” constrained to 3 because 73 of the 82 genera occur in fewer than three areas.

## Results

### Phylogeny and divergence times

The maximum clade credibility tree generated by BEAST analyses is well-resolved (Figure S1). Within the phaseoloid legumes, eight major clades were recognized, and the *Apios* is the earliest-diverging lineage (*PP* = 0.97). Psoraleeae and Desmodieae are strongly supported as monophyletic, both of which are imbedded within Phaseoleae.

**Figure 1:**
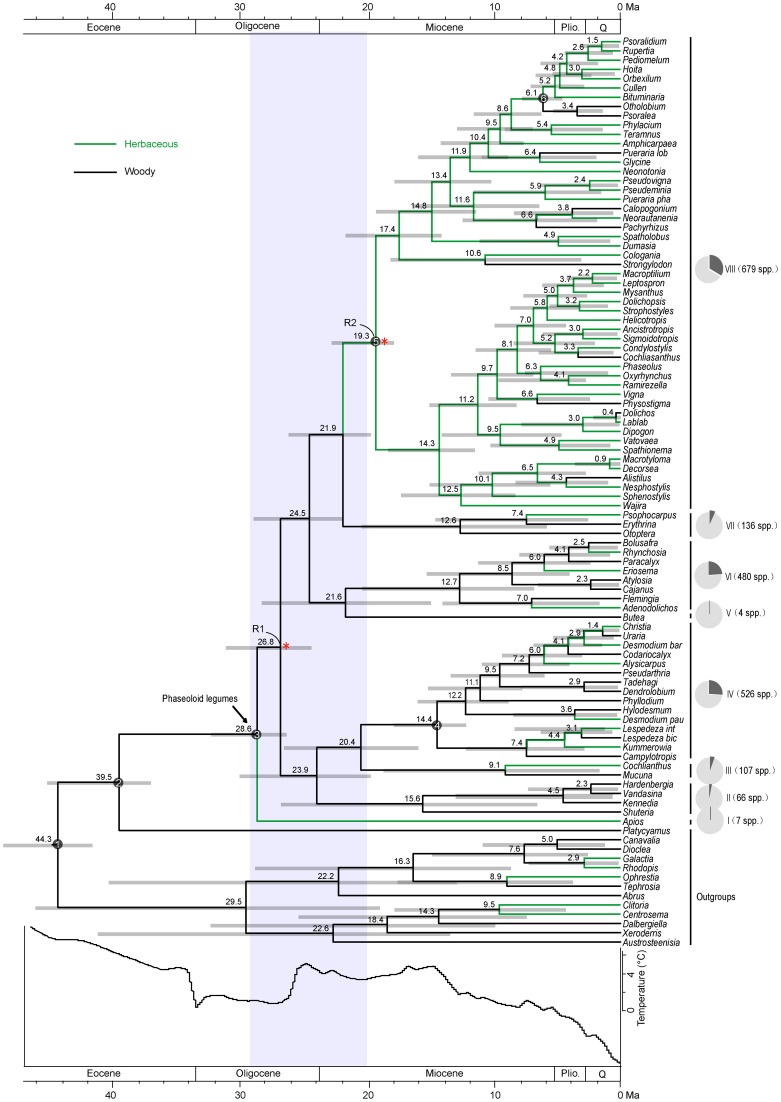
**Combined chronogram and habit shift analysis of the phaseoloid legumes**. Numbers above branches are divergence times estimated using BEAST. Gray bars show 95% credibility interval. White numbered nodes represents the calibration node. Red asterisks indicate the nodes (R1, R2) with significant rate increases. Shaded area at the base of the tree highlights initial diversification event. Pie charts represent the proportion of extant phaseoloid species in each clade. The depiction of sea-surface temperature changes is modified from Zachos et al. (2001).

Molecular dating shows a stem age for the phaseoloid legumes of 39.5 Ma (35.7–43.2 Ma 95% highest posterior density, HPD) (Figure 1). The earliest diverged *Apios* lineage (clade I) separated with the remaining phaseoloids at 28.6 Ma (HPD: 25.8–31.2 Ma). The remaining phaseoloid splits into other seven clades (clade II to VII) between 26.8 and 20.4 Ma. The most recent common ancestor (MRCA) of *Pueraria phaseoloides* and *Pueraria lobata* emerged at ca. 13.4 Ma (HPD: 9.9–17.0 Ma). The Psoraleeae crown age is estimated at 6.1 Ma (HPD: 4.8–7.7 Ma).

### Habit evolution

Results of ancestral habit state reconstruction are shown in Figure 1. The ancestral state of growth habit in the phaseoloid legumes is woody. Within the eight early diverged clades, six are woody (clade II–VII), while clade I and clade VIII are herbaceous. The herbaceous growth habit has evolved at least ten times within the phaseoloids. Importantly, clade VIII is the largest herbaceous lineage with some derived woody species, taking up ca. Thirty percentage species of the phaseoloid legumes.

### Diversification rates

**Figure 2:**
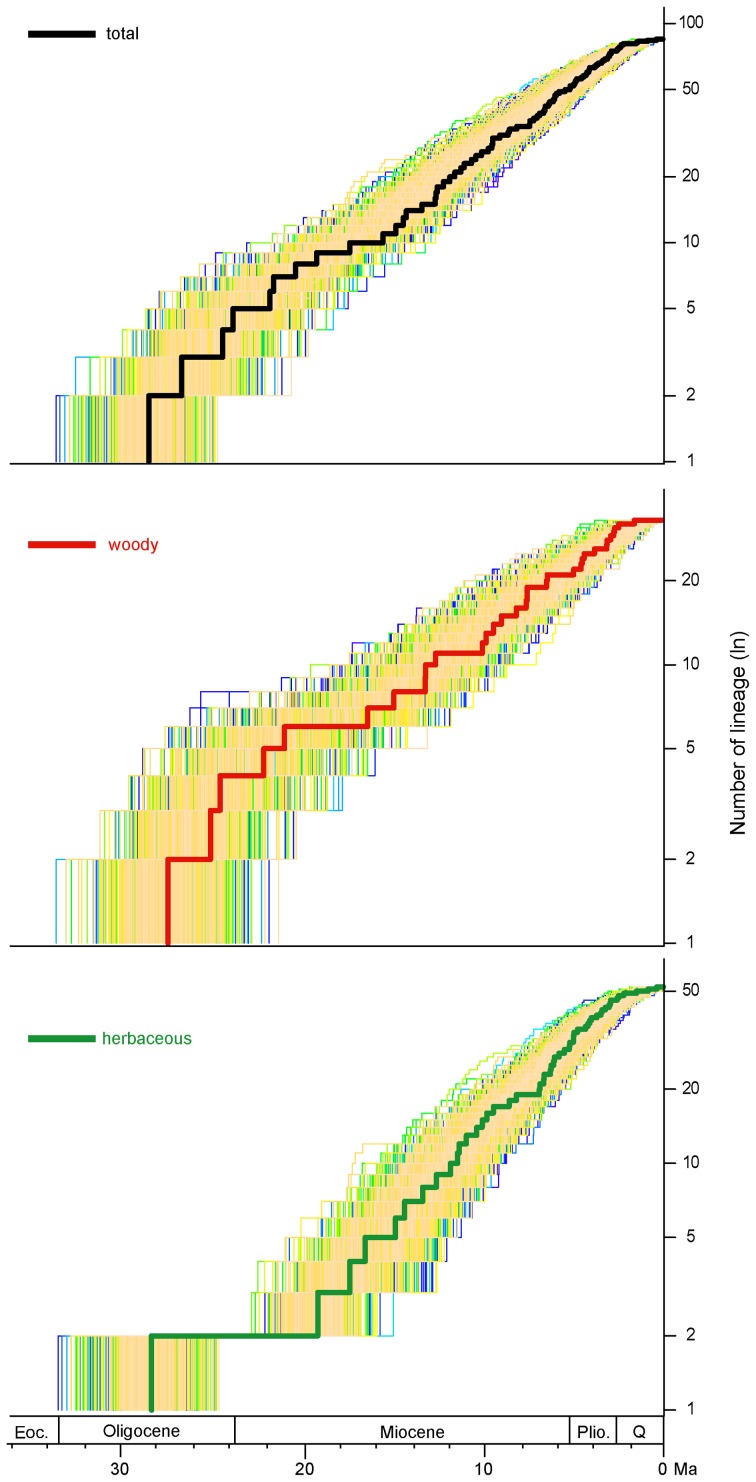
**Phaseoloid divergences through time, according to habit**.

The semilogarithmic lineage-through-time (LTT) plots for taxa of whole phaseoloid legumes, woody clades (clade II–VII) and herbaceous clades (clade I and VIII) are shown in Figure 2. The whole phaseoloid legumes and woody clades showed a high diversification rate at the early stages (20.4–28.6 Ma). Herbaceous lineages experienced a high diversification rate since the Early Miocene.

The relative cladogenesis (RC) statistic indicated two significant diversification rate shifts at node R1 and R2 within the phaseoloid legumes (Figure 1). The probability of R1 and R2 that had at least maximum descendents under the null hypothesis of a birth-death process are 0.024 and 0.010 separately (Table 1). Net diversification rates of the phaseoloid legumes were estimated as 0.13 (HPD: 0.12–0.15) net speciation events per million years (sp Myr^−1^) under a high relative extinction rate (ε = 0.9), and 0.19 sp Myr^−1^ under no extinction (ε = 0). Diversificaiton rates estimated for nodes R1 and R2 are shown in Table 2.

**Table 1:** **Relative cladogenesis (RC) test of the phaseoloid legumes. Nodes with diversification rate shift are shown on Figure 1**.

**Nodes**	Number of ancestor^*^	**Maximum descendents^†^**	***P*-value^‡^**
R1	2	84	0.024
R2	8	51	0.010

* Number of lineages alive before that node on Figure 1.

† Maximum number of descendents that node has at the present day based on Figure 1.

‡ The probability that node had at least maximum descendents under the null hypothesis of a birth-death process.

**Table 2:** **Net diversification rates (*r*) calculated for the phaseoloid legumes, nodes R1 and R2**.

**Clades**	**Stem group mean age (95%HPD)**	**No. species**	***r***			
			**ε= **0****	**95% HPD**	**ε = **0.9****	**95% HPD**
Phaseoloied legumes	39.5 (35.8–43.3)	2005	0.19	0.18–0.21	0.13	0.12–0.15
R1	28.6 (25.9–31.5)	1998	1.05	1.05–1.05	1.05	1.05–1.05
R2	21.9 (18.9–24.9)	679	0.30	0.26–0.35	0.19	0.17–0.22

### Biogeographical reconstruction

**Figure 3:**
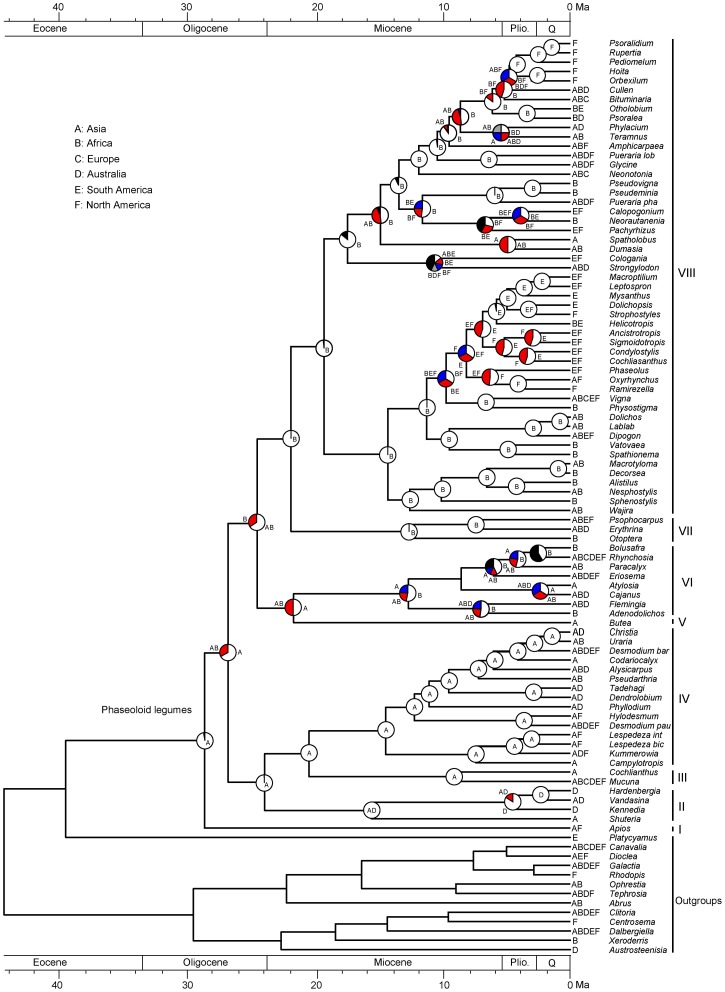
**Biogeographical reconstruction of the phaseoloid legumes**. The pie charts show the relative probabilities of alternative ancestral distributions obtained by Bayes-DIVA optimizations over the 1000 Bayesian trees (white > red). The first four areas with highest probability are colored according to relative probability in the following order: white > red > blue > gray; and the black portion represents reconstructions with a probability <0.10.

The results of ancestral area reconstruction using Bayes-DIVA in RASP is shown in Figure 3. The most recent common ancestor of the phaseoloid legumes is in Asia. Two independent intercontinental dispersal events occurred in the Late Oligocene. The first dispersal is to Africa with the rise of clade V, VI, VII, and VIII. The second dispersal is to Australia giving rise to clade II including *Hardenbergia,Vandasina,Kennedia*, and *Shuteria*. In the Miocene, the ancestral range of clade VIII expanded to South and North America following multiple dispersal events.

## Discussion

Our divergence time estimates (Figure 1) suggest a stem age of 39.5 Ma (HPD: 35.7–43.2 Ma) for the phaseoloid legumes and a crown age of 28.6 Ma (HPD: 25.8–31.2 Ma), which are consistent with the estimates of Lavin et al. (2005); Stefanović et al. (2009). Egan and Crandall (2008) estimate the crown age of 6.78 Ma for Psoraleeae, which is in accordance with our result (6.1 Ma; HPD: 4.8–7.7 Ma). Based on our time estimates, the MRCA of *Pueraria loata* and *Pueraria phaseoloides* dated back to the Middle Miocene (ca. 13.8 Ma), which coincides with the oldest fruit and foliage fossil records of *Pueraria* (Wang et al., 2010, and References therein). These suggest that our divergence date estimates for the phaseoloid legumes are reliable.

The phaseoloid legumes originated in the Late Eocene, but the group became differentiated in the Late Oligocene of Asia, and a dramatic accumulation of the phaseoloid lineages immediately occurred during the Late Oligocene and Early Miocene, with two dispersal events, from Asia to Africa and Australia. The rapid divergence time post-dates a period of a drastic global cooling resulting, in part, from the development of permanent continental ice-sheets in Antarctica (Zachos et al., 2001). This cooling induced a drier climate on a global scale (Zachos et al., 2001). The Himalaya-Tibetan plateau experienced rapid uplift at ~40 Ma (Zhang et al., 2006). These events fragmented the pan-Old World Eocene forest ecosystems and subsequently opened many new niches (Raup and Sepkoski, 1986; Prothero, 1994; Crisp and Cook, 2009). The RC test found that node R1 originated at 28.54 Ma (HPD: 25.76–31.23 Ma) had a significant rate increase (Figure 1; Table 1). Net diversification rate of node R1 is obviously higher than that of the whole phaseoloid legumes (Table 2). Considering the inferred credibility intervals of the estimated times of divergence, the eight early-divergent clades (I–VIII) seem to have occurred within an 8-million-year time window (28.6–20.4 Ma), which is a period of global warming emerged (Mosbrugger, 2005). Among the eight early-divergent clades, at least six is woody (Figure 1). Recent studies have indicated that shrubs and lianas can take advantage of some ecological opportunities and accordingly are regarded as early successional pioneer species (Shaver et al., 1997; Sturm et al., 2005; Tape et al., 2006; Bunn et al., 2007; Hallinger et al., 2010; Hallinger and Wilmking, 2011). Furthermore, the vigor and recruitment of shrubs and lianas can be enhanced by warming climate (Lantz et al., 2009; Forbes et al., 2010). Most of species in the six early-diverging woody clades are (sub)shrubs or lianas (Figure 1; Table S1). Four genera in woody phaseoloid clades, *Erythrina* (clade VII), *Flemingia* (clade VI), *Cajanus* (clade VI), and *Lespedeza* (clade IV), have been listed as the inclusion of invasive alien species (Rejmánek and Richardson, 2013). Even though our sampling is incomplete, the available morphological, polynological and molecular evidence suggests that species missing from our study would probably not fall within the stems of these woody clades (Table S1). Thus, our finding suggests that the early rapid diversification of the phaseoloid legumes was driven by ecological opportunities created by the emergence of new niches and range expansion, and the Late Oligocene global warming.

LTT plots indicate that the dramatic accumulation of the herbaceous phaseoloid lineages (clade VIII) occurred since the Early Miocene (Figure 2). The RC test found the other shift of diversification rates within the haseoloid legumes, node R2 (clade VIII), whose net diversification rate is higher than that of the whole phaseoloid legumes (Table 2). Clade VIII is herbaceous and contains about 52 genera and 679 species, greatly contributing to phaseoloid diversity (Figure 1). Our biogeographical reconstruction suggests that the MRCA of clade VIII is in Africa and subsequently multiple independent migrations from Africa to Asia, North America, and South America occurred (Figure 3). The clade VIII became diversified in the Early Miocene (19.3 Ma; HPD: 17.0–21.6 Ma), which corresponds to the time when the African plate collided with the Eurasian one (ca. 18–17 Ma; Axelrod and Raven, 1978). The geologic event resulted in the closure of the Tethys Sea and brought an end to the moist influence of the latitudinal oceanic circulation system (Axelrod and Raven, 1978; Jacobs, 2004), which, as well as higher global temperatures, may have induced a period of marked aridity in Africa. Moreover, Asia and America also experienced an analogous arid period (Guo et al., 2002; Minnich, 2007; Graham, 2010). The extensive aridity can have promoted the diversification of some groups inhabiting in dry regions, such as *Bursera* (De-Nova et al., 2012) and the ivesioids of *Potentilla* (Töpel et al., 2012). De-Nova et al. (2012) postulated that *Bursera* diversification during the Miocene might be related to the expansion of Mesoamerican seasonally dry tropical forests. The majority of clade VIII species are inhabited in seasonally dry tropical forests (Table 2). Thus, enhanced aridity, as well as the expansion of seasonally dry tropical forests in Africa, Asia, and America, would have promoted the diversification of the herbaceous phaseoloid legumes. Contrary to woody life forms, herbs have a shorter generation time, which will produce higher per-year mutation rates, thus increasing the genetic divergence and increasing speciation rates, consequentially making herbaceous lineages more diverse than woody plants (Eriksson and Bremer, 1992; Dodd et al., 1999; Verdú, 2002). Thus, a habit shift from woody to herbaceous may have acted as a key innovation that resulted in an increased diversification rate of the phaseoloid legumes in the Miocene. Additionally, some species of the phaseoloid legumes are distributed in grasslands (Table 1; Lewis et al., 2005), which may be associated with the development of grassland ecosystems during the Late Cenozoic (Strömberg, 2011). Nevertheless, a species-level taxon sampling will be needed to examine whether the shift of habitats have also been responsible for diversification of clade VIII.

## Author contributions

Wei Wang and Zhiduan Chen conceived the study. Honglei Li performed the experiments. Honglei Li, Wei Wang, Li Lin, and Xinyu Zhu analyzed the data. Wei Wang, Jianhua Li, Xiangyun Zhu, and Zhiduan Chen contributed reagents/materials/analysis tools. Honglei Li and Wei Wang wrote the paper. Wei Wang, Jianhua Li, and Zhiduan Chen edited the paper.

### Conflict of interest statement

The authors declare that the research was conducted in the absence of any commercial or financial relationships that could be construed as a potential conflict of interest.
