# Supplementary material for: Diversification of the phaseoloid legumes: effects of climate change, range expansion and habit shift
Source: Front Plant Sci. 2013 Oct 9;4:386. doi: 10.3389/fpls.2013.00386 (PMC3793175; doi:10.3389/fpls.2013.00386)
Supplement: Supplementary file 1 [file DataSheet1.DOC]

**Supplementary Material**

**Table S1. Genera of the phaseoloid legumes indicating the number of recognized species, habits, geographic distributions, and inhabited biomes.**

**Table S2. Taxa, vouchers and GenBank accession numbers for the sequences used in this study.**

**Table S3. Primers used for amplification and sequencing in this study.**

**Figure S1. Phylogeny of the phaseoloid legumes.**

**Tables S1-S3 and Figure S1 Legends**

Table S1**.** Genera of the phaseoloid legumes indicating the number of recognized species, habits, geographic distributions, and inhabited biomes. “*” indicates those genera not sampled in this study, whose close relatives are justified based on the literature. Clades I - VIII is based on Lewis *et al.* (2005) and this study.

Table S2**.** Taxa, vouchers and GenBank accession numbers for the sequences used in this study. “-” indicates missing data . “*” indicates newly generated sequences in this study.

Table S3**.** Primers used for amplification and sequencing in this study.

**Figure S1.** Phylogeny of the phaseoloid legumes using BEAST. Numbers above the branches are posterior probability values (> 0.5).

**Table S1**

| Clade | Genus | No. species | Habit | Distribution | Biome | Justification |
| --- | --- | --- | --- | --- | --- | --- |
| I | *Apios* | 7 | herb | Asia, North America | subtropical to temperate forest and grassland |  |
| II | *Diphyllarium** | 1 | herb or liana | Asia | seasonally dry tropical forest | Based on climbing habit and presence of bracteoles, *Diphyllarium* is close to *Shuteria* (Lackey, 1981) |
|  | *Hardenbergia* | 3 | herb or subshrub | Australia | dry to wet sclerophyll forest |  |
|  | *Kennedia* | 15 | herb or subshrub | Australia | seasonally dry tropical to temperate forest |  |
|  | *Mastersia** | 2 | herb or liana | Asia | seasonally dry tropical forest | Based on climbing habit and bracteoles shorter than calyx, *Mastersia* is close to *Shuteria* (Lackey, 1981) |
|  | *Shuteria* | 44 | herb or liana | Asia | seasonally dry tropical to subtropical forest |  |
|  | *Vandasina* | 1 | herb or liana | Asia, Australia | seasonally dry tropical forest and grassland |  |
| III | *Cochlianthus* | 2 | herb | Asia | temperate forest and grassland |  |
|  | *Mucuna* | 105 | liana | worldwide | tropical rain forest to seasonally dry forest |  |
| IV | *Akschindlium** | 1 | (sub)shrub | Asia | seasonally dry tropical to subtropical forest and grassland | Based on axillary stamen-filaments free or slightly connate at base to others, *Akschindlium* is close to *Tadehagi* (Ohashi, 2003) |
|  | *Alysicarpus* | 27 | herb | Africa, Asia, Australia | seasonally dry tropical forest and grassland |  |
|  | *Aphyllodium** | 7 | (sub)shrub | Asia | seasonally dry tropical forest and grassland | Based on campanulate calyx and indehiscent fruit, *Aphyllodium* is close to *Desmodium* (Ohashi, 2004) |
|  | *Arthroclianthus** | 30 | shrub or tree | Europe | seasonally dry tropical forest | Based on molecular analysis, *Arthroclianthus* is close to *Trifidacanthus* ( Bailey et al., 1997) |
|  | *Campylotropis* | 37 | shrub | Asia | seasonally dry tropical to temperate forest |  |
|  | *Christia* | 10 | herb | Asia, Australia | seasonally dry tropical forest and grassland |  |
|  | *Codariocalyx* | 2 | shrub | Asia | seasonally dry tropical forest and grassland |  |
|  | *Dendrolobium* | 18 | herb, shrub or tree | Asia, Australia | seasonally dry tropical forest or grassland |  |
|  | *Desmodiastrum** | 4 | herb | Asia | seasonally dry tropical grassland | Based on not inflated fruits, *Desmodiastrum* is close to *Alysicarpus* (Ohashi et al., 1981) |
|  | *Desmodium* | 275 | herb, shrub or tree | Asia, Australia, North America, South America, Africa | seasonally dry to wet tropical, temperate forest and grassland |  |
|  | *Droogmansia** | 5 | (sub)shrub | Africa | seasonally dry tropical forest and grassland | Based on winged petioles and 1-foliolate, *Droogmansia* is close to *Tadehagi* (Ohashi et al., 1981) |
|  | *Eleiotis** | 2 | herb | Asia | seasonally dry tropical forest | Based on indehiscent fruit, *Eleiotis* is close to *Phyllodium* (Ohashi et al., 1981) |
|  | *Hanslia** | 2 | shrub | Asia | seasonally dry to wet tropical forest | Based on distinctly jointed fruits, *Hanslia* is close to *Desmodium* (Ohashi et al., 1981) |
|  | *Hegnera** | 1 | subshrub | Asia | seasonally dry tropical forest | Based on distinctly jointed fruits, *Hegnera* is close to *Desmodium* (Ohashi et al., 1981) |
|  | *Hylodesmum* | 14 | herb or subshrub | Asia, North America | tropical to temperate forest and grassland |  |
|  | *Kummerowia* | 2 | herb | Asia, North America, Australia | temperate grassland |  |
|  | *Leptodesmia** | 3 | herb or subshrub | Asia, Africa | seasonally dry tropical forest and grassland | Based on complex granular interstitium in pollen, *Leptodesmia* is close to *Uraria* (Ferguson and Skvarla, 1983) |
|  | *Lespedeza* | 35 | herb or (sub)shrub | Asia, North America | seasonally dry tropical to temperate forest and grassland |  |
|  | *Mecopus** | 1 | herb or subshrub | Asia | seasonally dry tropical forest and grassland | Based on not accrescent calyx, *Mecopus* is close to *Uraria* (Ohashi et al., 1981) |
|  | *Melliniella** | 1 | herb | Africa | seasonally dry tropical grassland | Based glumaceous calyx, *Melliniella* is close to *Alysicarpus* (Ohashi et al., 1981) |
|  | *Monarthrocarpus** | 1 | shrub | Asia, Australia | seasonally dry tropical forest | Based on distinctly jointed fruits, *Monarthrocarpus* is close to *Desmodium* (Ohashi et al., 1981) |
|  | *Nephrodesmus** | 6 | shrub or tree | Europe | seasonal tropical forest | Based on spines lacking and bracteoles present, *Nephrodesmus* is close to *Trifidacanthus* (Ohashi et al. 1981) |
|  | *Ohwia** | 2 | shrub | Asia | seasonally dry tropical forest and grassland | Based on distinctly jointed fruits, *Ohwia* is close to *Desmodium* (Ohashi et al., 1981) |
|  | *Ougeinia** | 1 | tree | Asia | seasonally dry tropical forest | Based on distinctly jointed fruits, *Ougeinia* is close to *Desmodium* (Ohashi et al., 1981) |
|  | *Phyllodium* | 8 | shrub | Asia, Australia | seasonally dry tropical forest and grassland |  |
|  | *Pseudarthria* | 3 | herb or (sub)shrub | Africa, Asia | seasonally dry tropical forest and grassland |  |
|  | *Pycnospora** | 1 | herb or subshrub | Africa, Asia, Australia | seasonally dry tropical forest and grassland | Based on not looped pedicels, *Pycnospora* is close to *Uraria* (Ohashi et al., 1981) |
|  | *Tadehagi* | 6 | (sub)shrub | Asia, Australia | seasonally dry tropical to subtropical forest and grassland |  |
|  | *Trifidacanthus** | 1 | shrub or tree | Asia | seasonally dry tropical forest and grassland | Based on upper (adaxial) suture slightly con­stricted or straight and lower (abaxial) one constricted of the fruit, Ohashi et al. (1996) concluded *Trifidacanthus* is close to *Desmodium* |
|  | *Uraria* | 20 | (sub)shrub | Asia, Africa | seasonally dry tropical forest and grassland |  |
| V | *Butea* | 2 | tree or liana | Asia | seasonally dry tropical forest and grassland |  |
|  | *Meizotropis** | 2 | shrub | Asia | seasonally dry tropical forest and grassland | Based on the colpus ends and ornamentation of pollens, *Meizotropis* is close to *Butea* (Ridder-Numan and van der Ham, 1997) |
| VI | *Adenodolichos* | 3 | herb or shrub | Africa | seasonally dry tropical forest and grassland |  |
|  | *Atylosia* | 1 | shrub | Asia | seasonally dry tropical forest and grassland |  |
|  | *Bolusafra* | 1 | herb or subshrub | Africa | mediterranean forest |  |
|  | *Cajanus* | 33 | herb or shrub | Asia, Australia, Africa | seasonally dry tropical forest and grassland |  |
|  | *Carrissoa** | 1 | subshrub | Africa | seasonally dry tropical forest | Based on not expanded calyx-lobes and pinnately compound leaves, *Carrissoa* is close to *Rhynchosia* (Lackey, 1981) |
|  | *Chrysoscias** | 3 | herb or subshrub | Africa | mediterranean sclerophyllous forest | Based on funicle attached to the hilum in the middle, *Chrysoscias* is close to *Rhynchosia* (Moteetee et al., 2012) |
|  | *Dunbaria** | 20 | herb or subshrub | Asia, Australia | seasonally dry tropical forest and grassland | Based on compressed fruits, *Dunbaria* is close to *Cajanus* (Lackey, 1981) |
|  | *Eriosema* | 150 | herb or subshrub | Asia, Australia, North America, South America, Africa | seasonally dry tropical to subtropical forest and grassland |  |
|  | *Flemingia* | 32 | herb or shrub | Asia, Australia, Africa | seasonally dry tropical forest and grassland |  |
|  | *Paracalyx* | 6 | herb | Africa, Asia | seasonally dry tropical forest |  |
|  | *Rhynchosia* | 230 | herb or subshrub | world wide | seasonally dry forest and grassland |  |
| VII | *Dysolobium** | 4 | herb or liana | Asia | seasonally dry tropical forest | Based on seeds with a shorter hilum, *Dysolobium* is close to *Psophocarpus* (Lackey, 1981) |
|  | *Erythrina* | 120 | tree or shrub | Asia, Australia, Africa | seasonally dry tropical to subtropical forest and grassland |  |
|  | *Otoptera* | 2 | herb or shrub | Africa | seasonally dry tropical forest and grassland |  |
|  | *Psophocarpus* | 10 | herb or subshrub | Asia, North America, South America, Africa | seasonally dry tropical forest and grassland |  |
| VIII | *Alistilus* | 3 | herb or subshrub | Africa | seasonally dry tropical forest and grassland |  |
|  | *Amphicarpaea* | 4 | herb | Asia, North America, Africa | seasonally dry tropical forest and grassland |  |
|  | *Ancistrotropis* | 6 | herb | North America, South America | seasonally dry tropical forest |  |
|  | *Austrodolichos** | 1 | herb | Australia | seasonally dry tropical forest and grassland | Based on stamen-filaments dilated above and vexillary stamen with a hook at the base, *Austrodolichos* is close to *Nesphostylis* (Lackey, 1981) |
|  | *Bituminaria* | 2 | herb | Europe, Asia, Africa | mediterranean forest and grassland |  |
|  | *Calopogonium* | 5 | herb or subshrub | North America, South America | seasonally dry tropical to subtropical forest |  |
|  | *Cochliasanthus* | 1 | herb | North America, South America | seasonally dry tropical forest |  |
|  | *Cologania* | 12 | herb | North America, South America | tropical to temperate forest and grassland |  |
|  | *Condylostylis* | 4 | herb | North America, South America | seasonally dry tropical forest |  |
|  | *Cullen* | 34 | herb, shrubshrub or tree | Asia, Australia, Africa | seasonally dry tropical to temperate forest, grassland and desert |  |
|  | *Decorsea* | 6 | herb or subshrub | Africa | seasonally dry tropical to substropical forest |  |
|  | *Dipogon* | 1 | herb | Asia, North America, South America, Africa | temperate and mediterranean forest |  |
|  | *Dolichopsis* | 1 | herb | South America | seasonally tropical to subtropical grassland |  |
|  | *Dolichos* | 60 | herb or subshrub | Asia, Africa | seasonally dry tropical to subtropical forest and grassland |  |
|  | *Dumasia* | 10 | herb | Asia, Africa | seasonally dry tropical to temperate forest |  |
|  | *Eminia** | 4 | herb or shrub | Africa | seasonally dry tropical forest and grassland | Based on two flowers per node of the inflorescence, *Eminia* is close to *Pseudeminia* (Lackey, 1981) |
|  | *Glycine* | 19 | herb | Asia, Australia, North America, Africa | seasonally dry tropical to temperate forest and grassland |  |
|  | *Helicotropis* | 4 | herb | South America, Africa | seasonally dry to wet forest and grassland |  |
|  | *Herpyza** | 1 | herb | North America | seasonally dry tropical forest and grassland | Based on separate uper calyx-lobes, *Herpyza* is close to *Calopogonium* (Lackey, 1981) |
|  | *Hoita* | 3 | herb | North America | mediterranean forest and grassland |  |
|  | *Lablab* | 1 | herb | Asia, Africa | seasonally dry tropical to subtropical forest and grassland |  |
|  | *Leptospron* | 2 | herb | North America, South America | secondary and primary forest |  |
|  | *Macroptilium* | 17 | herb | North America, South America | seasonally dry tropical to subtropical forest and grassland |  |
|  | *Macrotyloma* | 24 | herb | Asia, Africa | seasonally dry tropical to subtropical forest and grassland |  |
|  | *Mysanthus* | 1 | herb | South America | seasonally dry tropical forest and grassland |  |
|  | *Neocollettia** | 1 | herb | Asia | seasonally dry tropical forest and grassland | Based on molecular analysis, *Neocollettia* is close to *Pueraria stricta* (Lee and Hymowitz, 2001) |
|  | *Neonotonia* | 2 | herb | Africa, Asia, Europe | seasonally dry tropical forest and grassland |  |
|  | *Neorautanenia* | 3 | herb or subshrub | Africa | seasonally dry tropical forest and grassland |  |
|  | *Nesphostylis* | 4 | herb | Asia, Africa | seasonally dry tropical to subtropical forest and grassland |  |
|  | *Nogra** | 3 | herb | Asia | seasonally dry tropical forest | Based on molecular analysis, *Nogra* is close to *Pueraria* *montana* (Lee and Hymowitz, 2001) |
|  | *Orbexilum* | 8 | herb | North America | temperate forest and grassland |  |
|  | *Oryxis** | 1 | herb or subshrub | South America | seasonally dry tropical forest and grassland | Based on campanulate calyx and linear-oblong fruit, *Oryxis* is close to *Dolichopsis* (Delgado and Lewis,1997) |
|  | *Otholobium* | 61 | herb, shrub or tree | South America, Africa | seasonally dry tropical to mediterranean forest and grassland |  |
|  | *Oxyrhynchus* | 4 | herb or shrub | North America, Asia | seasonally dry to wet tropical and subtropical forest |  |
|  | *Pachyrhizus* | 5 | herb or subshrub | North America, South America | seasonally dry tropical forest |  |
|  | *Pediomelum* | 21 | herb | North America | temperate forest, grassland and desert |  |
|  | *Phaseolus* | 62 | herb | North America, South America | seasonally dry to wet forest, grassland and desert |  |
|  | *Phylacium* | 2 | herb | Asia, Australia | seasonally dry tropical forest and grassland |  |
|  | *Physostigma* | 4 | herb or subshrub | Africa | seasonally dry to wet tropical forest grassland |  |
|  | *Pseudeminia* | 4 | herb or subshrub | Africa | seasonally dry tropical forest and grassland |  |
|  | *Pseudovigna* | 2 | herb | Africa | seasonally dry tropic forest and grassland |  |
|  | *Psoralea* | 50 | herb, shrub or tree | Australia, Africa | seasonally dry tropical to mediterranean forest and grassland |  |
|  | *Psoralidium* | 3 | herb | North America | temperate forest, grassland and desert |  |
|  | *Pueraria* | 18 | herb, shrub or liana | Asia, Australia, North America, Africa | seasonally dry tropical to subtropical forest and rain forest |  |
|  | *Ramirezella* | 7 | herb | North America | seasonally dry tropical forest |  |
|  | *Rupertia* | 3 | herb | North America | temperate to mediterranean forest and grassland |  |
|  | *Sigmoidotropis* | 9 | herb | North America, South America | seasonally dry tropical forest |  |
|  | *Sinodolichos** | 2 | herb | Asia | seasonally dry tropical forest | Based on molecular analysis, *Sinodolichos* is close to *Pseudeminia* (Doyle et al., 2003) |
|  | *Spathionema* | 1 | subshrub | Africa | seasonally dry tropical forest, grassland and desert |  |
|  | *Spatholobus* | 29 | liana | Asia | seasonally dry to evergreen tropical forest |  |
|  | *Sphenostylis* | 7 | herb or subshrub | Africa | seasonally dry tropical to subtropical forest and grassland |  |
|  | *Strongylodon* | 12 | liana | Asia, Australia, Africa | tropical rain forest |  |
|  | *Strophostyles* | 3 | herb | North America | subtropical to temperate forest and grassland |  |
|  | *Teramnus* | 9 | herb or subshrub | Asia, Africa | seasonally dry tropical forest, grassland |  |
|  | *Teyleria** | 3 | herb | Asia | seasonally dry tropical forest | Based on molecular analysis, *Teyleria* is close to *Neonotonia* (Doyle et al., 2003) |
|  | *Vatovaea* | 1 | shrub | Africa | seasonally dry tropical forest, grassland and semi-desert |  |
|  | *Vigna* | 104 | herb | Asia, North America, South America, Africa, Europe | seasonally dry tropical forest and grassland |  |
|  | *Wajira* | 5 | herb or subshrub | Asia, Africa | seasonally dry tropical forest and grassland |  |

Data are obtained from several sources (White, 1983; Tiffney and Mazer, 1995; Verdú, 2002; Sprent, 2007; Electronic databases: [www.efloras.org](http://www.efloras.org/)).

**Table S2**

| Taxon | Voucher | *trnL-F* | *rbcL* | *trnK/matK* |
| --- | --- | --- | --- | --- |
| *Abrus precatorius* | ^1^Thorne et al. 6791 (BRY);  ^2^Doyle 1291 (BH); ^3^Hu 1136 (USDA) | ^1^EF543423 | ^2^U74224 | ^3^AF142705 |
| *Adenodolichos rupestris* | Potter 870418–01 (BH) | - | AF308700 | - |
| *Alistilus jumellei* | Thulin & Razafindraibe 11823 (UPS) | - | - | JN008191 |
| *Alysicarpus vaginalis* | M. Mikage et al. 9554173 (E) | - | JQ933214 | JQ587508 |
| *Amphicarpaea bracteata* | Doyle 1132 (BH) | EU717317 | EU717257 | EU717399 |
| *Ancistrotropis peduncularis* | Salas 4259 (MEXU) | - | - | JN008272 |
| *Apios americana* | Yatskievych & McCray 86129 (IND) | EU717312 | EU717285 | EU717426 |
| *Atylosia lineata* | Doyle 1288 (BH) | EU717308 | AF181931 | - |
| *Austrosteenisia blackii* | Pedley 5005 (K) | AF311381 | U74242 | AF142707 |
| *Bituminaria bituminosa* | NF Weeden s.n. (BH) | EU717349 | EU717255 | EU717398 |
| *Bolusafra bituminosa* | Doyle 1282 (BH) | EU717309 | EU717272 | EU717413 |
| *Butea monosperma* | ^1^Ridder-Numan s.n. (L) | - | JX141401 | ^1^JN008175 |
| *Cajanus cajan* | Stefanović SS0302 (TRTE) | EU717310 | EU717273 | EU717414 |
| *Calopogonium caeruleum* | Doyle 1294 (BH) | EU717318 | AF308723 | - |
| *Campylotropis macrocarpa* | StefanovićSS0304 (TRTE) | EU717298 | EU717277 | EU717418 |
| *Canavalia ensiformis* | Doyle 1107 (BH) | EU717354 | U74238 | - |
| *Centrosema sagittatum* | iBOL Working Group BioBot00292 (2012, unpublished) | - | JQ591640 | JQ587552 |
| *Christia vespertilionis* | Su JX & Lu LM102 (PE) | KF621108* | KF621119* | KF621098* |
| *Clitoria ternatea* | Doyle 1507 (BH) | EU717355 | EU717286 | EU717427 |
| *Cochlianthus gracilis* | Zhu XY 1471 (PE) | KF621109* | KF621120* | - |
| *Cochliasanthus caracalla* | Caballero-Pardo 50 (MEXU) | - | - | JN008274 |
| *Codariocalyx motorius* | Zhu XY 2009083-2 (PE) | KF621110* | KF621121* | KF621099* |
| *Cologania lemmonii* | Mendoza 16933 (MEXU) | EU717319 | EU717264 | EU717405 |
| *Condylostylis venusta* | CIAT 2468 (MONT) | - | - | JN008261 |
| *Cullen australasicum* | Adams et al. 1999 (Stefanović et al., 2009) | EU717350 | EU717254 | EU717397 |
| *Dalbergiella nyasae* | ^1^Lavin s.n. (K); ^2^Muller 2686 (K) | - | ^1^AF308724 | ^2^AF142706 |
| *Decorsea schlechteri* | National Botanic Gardens Belgium accession X2915 (Thulin et al., 2004) | - | - | AY582975 |
| *Dendrolobium dispermum* | Y. Iokawa 5393 (Nemoto et al., 2010) | AB538884 | - | - |
| *Desmodium barbatum* | SRPIS227476 (Stefanović et al., 2009) | EU717290 | EU717279 | EU717420 |
| *Desmodium pauciflorum* | Stefanović SS0327 (TRTE) | EU717297 | EU717280 | EU717421 |
| *Dioclea malacocarpa* | iBOL Working Group BioBot06020 (2012, unpublished) | - | JQ591714 | JQ587606 |
| *Dipogon lignosus* | J. Gibson 145 (UCD) | - | AB045800 | AY582988 |
| *Dolichopsis paraguariensis* | Krapovickas 46512 (MEXU) | - | - | AY509942 |
| *Dolichos lablab* | Cultivated (IUGH) | EU717339 | EU717267 | EU717408 |
| *Dumasia villosa* | Doyle 1491 (BH) | EU717320 | EU717265 | EU717406 |
| *Eriosema himalaicum* | Zhu XY 2009105-1 (PE) | KF621111* | KF621122* | KF621100* |
| *Erythrina sousae* | Bruneau 455 (BH) | EU717313 | EU717270 | EU717411 |
| *Flemingia macrophylla* | Su JX & Lu LM 206 (PE) | KF621112* | KF621123* | KF621101* |
| *Galactia striata* | Doyle 1130 (BH) | EU717356 | EU717287 | EU717428 |
| *Glycine max* | Lavin #72-15II94 (MONT) | EU717321 | EU717256 | AF142700 |
| *Hardenbergia violacea* | Doyle 1015 (BH) | EU717331 | EU717284 | EU717425 |
| *Helicotropis linearis* | Beck 24136 (MEXU) | - | - | JN008258 |
| *Hoita macrostachya* | Egan & Egan 271 (BRY) | EF543364 | - | EF549954 |
| *Hylodesmum laxum* | Zhu XY 0513 (PE) | KF621113* | KF621124* | KF621102* |
| *Kennedia nigricans* | DLEG920034 (Stefanović et al., 2009) | EU717335 | EU717283 | EU717424 |
| *Kummerowia stipulacea* | Stefanović SS04148 (TRTE) | EU717299 | EU717276 | EU717417 |
| *Lablab purpureus* | Lavin s.n. (MONT) | EU717339 | EU717267 | EU717408 |
| *Leptospron adenanthum* | CIAT 4022 (Delgado-Salinas et al., 2011) | - | - | JN008271 |
| *Lespedeza bicolor* | SRPIS286476 (Stefanović et al., 2009) | EU717301 | EU717274 | EU717415 |
| *Lespedeza intermedia* | StefanovićSS0325 (TRTE) | EU717304 | EU717278 | EU717419 |
| *Macroptilium atropurpureum* | Stefanović SS0306 (TRTE) | EU717340 | EU717268 | EU717409 |
| *Macrotyloma uniflorum* | SRPIS297892 (Stefanović et al., 2009) | EU717341 | EU717269 | EU717410 |
| *Mucuna cochinchinensis* | Zhu XY 15685 (PE) | KF621114* | KF621125* | KF621103* |
| *Mysanthus uleanus* | G. P. Lewis 1923 (MEXU) | - | - | AY509941 |
| *Neonotonia wightii* | Doyle 1295 (BH) | EU717323 | EU717261 | EU717402 |
| *Neorautanenia mitis* | Hedren et al. 566 (UPS) | - | AF308715 | JN008178 |
| *Nesphostylis holosericea* | Thoen 5 (K) | - | - | AY582979 |
| *Ophrestia radicosa* | Doyle 1514 (BH) | EU717359 | EU717289 | EU717430 |
| *Orbexilum lupinellum* | Egan & Egan 257 (BRY) | EF543365 | - | EF549995 |
| *Otholobium sericeum* | KLC 1986-2127 (K) | EU717351 | U74219 | - |
| *Otoptera burchellii* | Skarpe S-134 (UPS) | - | - | JN008176 |
| *Oxyrhynchus volubilis* | Cortez & Vazquez 195 (MEXU) | - | AF308717 | AY509935 |
| *Pachyrhizus erosus* | Kajita et al. 2001 (Stefanović et al., 2009) | EU717324 | EU717260 | EU717401 |
| *Paracalyx schweinfurthii* | A.G. Miller et al. M10145 (E) | - | JQ933431 | - |
| *Pediomelum argophyllum* | Egan & Egan 213 (BRY) | EF543377 | - | EF549988 |
| *Phaseolus coccineus* | Lavin s.n. (MONT) | GQ411559 | GQ411656 | DQ445966 |
| *Phylacium majus* | Kajita et al. (2000, unpublished) | - | AB045815 | - |
| *Phyllodium pulchellum* | PS0252MT01 (Gao et al., 2011) | - | - | HM049524 |
| *Physostigma venenosum* | C. C. H. Jongkind 1792 (MEXU) | - | - | AY582998 |
| *Platycyamus regnellii* | Brazil Lima s.n. (RB) | AF311378 | AB045817 | AF142709 |
| *Pseudarthria hookeri* | iBOL Working Group OM1473 (2012, unpublished) | - | JF265559 | JF270902 |
| *Pseudeminia comosa* | Doyle 1923 (BH) | - | AF181936 | - |
| *Pseudovigna argentea* | Doyle 1021 (BH) | EU717325 | EU717262 | EU717403 |
| *Psophocarpus tetragonolobus* | SRPIS477254 (Stefanović et al., 2009) | EU717343 | EU717271 | EU717412 |
| *Psoralea aphylla* | B & T World Seeds 35039 (Stefanović et al., 2009) | EU717352 | - | - |
| *Psoralidium lanceolatum* | Egan & Egan 150 (BRY) | EF543355 | - | EF549941 |
| *Pueraria lobata* | Doyle 1421 (BH) | EU717326 | EU717259 | - |
| *Pueraria phaseoloides* | Doyle 110 (BH) | EU717327 | EU717263 | EU717404 |
| *Ramirezella strobilophora* | Perez J. s.n. (MEXU) | EU717344 | - | AY509936 |
| *Rhodopis planisiliqua* | Zanoni et al. 43304 (MO) | - | AF308728 | - |
| *Rhynchosia himalensis var. craibiana* | STE team 401 (PE) | KF621115* | KF621126* | KF621104* |
| *Rupertia hallii* | Egan & Egan 277 (BRY) | EF543348 | - | EF549953 |
| *Shuteria vestita* | Doyle 1114 (BH) | EU717328 | EU717282 | EU717423 |
| *Sigmoidotropis ampla* | Quintana Roo CIAT 4070 (Delgado-Salinas et al., 2011) | - | - | JN008240 |
| *Spathionema kilimandscharicum* | Pasquet 1063 (K) | - | - | AY582990 |
| *Spatholobus sp.* | Zhu XY 2009068-2 (PE) | KF621116* | - | KF621105* |
| *Sphenostylis angustifolia* | Van Wyk 2604 (BH) | - | - | AY582978 |
| *Strongylodon macrobotrys* | Bruneau974 (BH) | - | AF308729 | - |
| *Strophostyles helvola* | Pittman et al. s.n. (USCH) | EU717345 | - | DQ443469 |
| *Tadehagi triquetrum* | Zhu XY 2009052-1 (PE) | KF621117* | KF621127* | KF621106* |
| *Tephrosia rhodesica* | Cultivated (IUGH) | EU717360 | EU717288 | EU717429 |
| *Teramnus uncinatus* | Cultivated (IUGH) | EU717330 | EU717258 | EU717400 |
| *Uraria lagopodioides* | Zhu XY 2009042-1 (PE) | KF621118* | KF621128* | KF621107* |
| *Vandasina retusa* | Uhl 602 (BH) | EU717338 | - | - |
| *Vatovaea pseudolablab* | Harrar, Gilbert & Thulin 136 (UPS) | - | - | AH013772 |
| *Vigna unguiculata* | J.C. Baudet 114 (MONT) | EU717348 | EU717266 | EU717407 |
| *Wajira grahamiana* | Pasquet 1051 (EA) | - | - | AY583005 |
| *Xeroderris stuhlmannii* | Corloy2162 (K) | - | AF308727 | AF142708 |

**Table S3**

| Primer | | Sequence (5’-3’) | Reference |
| --- | --- | --- | --- |
| *rbcL* | *rbcL*-NF | ATGTCACCACAAACAGAAAC | This study |
|  | *rbcL*-NR | CTAGTTATCCATTGCTGGG | This study |
|  | *rbcL*-27F | CAACTGTTGGGTTCAAAGC | This study |
|  | *rbcL*-1341R | AGACTTCACAAGCAGCAGC | This study |
| *trnL-F* | *trnL* | CGAAATCGGTAGACGCTACG | Taberlet et al., 1991 |
|  | *trnF* | ATI'TGAACTGGTGACACGAG | Taberlet et al., 1991 |
|  | *trnL*-XF | GGATATGGCGAAATTGGTAG | This study |
|  | *trnL*-MF | GGGATAGAGGGACTTGAACC | This study |
|  | *trnF*-MR | AGGAACCAGATTTGAACTGG | This study |
|  | *trnF*-XR | AGGAACCAGATTTGAACTGG | This study |
| *matK/trnK* | *trnK*685F | GTATCGCACTATGTATCATTTGA | Wojciechowski et al., 2004 |
|  | *matK*4La | CCTTCGATACTGGGTGAAAGAT | Wojciechowski et al., 2004 |
|  | *matK*1100L | TTCAGTGGTACGGAGTCAAATG | Wojciechowski et al., 2004 |
|  | *matK*1932Ra | CCAGACCGGCTTACTAATGGG | Wojciechowski et al., 2004 |
|  | *matK*832R | TTGCATAGAAATAGATTCGCTCAAA | Wojciechowski et al., 2004 |
|  | *trnK*2R | CCCGGAACTAGTCGGATGG | Wojciechowski et al., 2004 |

**Supplementary References**

Bailey, C.D., Doyle, J.J., Kajita, T., Nemoto, T., and Ohashi, H. (1997). The chloroplast *rpl2* intron and ORF184 as phylogenetic markers in the legume tribe Desmodieae. *Syst. Bot.* 22, 133-138.

Delgado, S. and Lewis, G.P. (1997). *Oryxis*, a new genus in tribe Phaseoleae (Leguminosae: Papilionoideae) from Brazil. *Kew Bull.* 52, 221-225.

Doyle, J.J., Doyle, J.L. and Harbison, C. (2003). Chloroplast-expressed glutamine synthetase in *Glycine* and related Leguminosae: phylogeny, gene duplication, and ancient polyploidy. *Syst. Bot.* 28, 567-577.

Ferguson, I.K. and Skvarla, J.J. (1983). The granular interstitium in the pollen of subfamily Papilionoideae (Leguminosae). *Am. J. Bot.* 70, 1401-1408.

Lackey, J.A. (1981). “Phaseoleae” in *Advances in Legume Systematics, Part 1.* eds. R.M. Polhill, and P.H. Raven (Kew: Royal Botanic Gardens), 301-327.

Lee, J., and Hymowitz, T. (2001). A molecular phylogenetic study of the subtribe Glycininae (Leguminosae) derived from the chloroplast DNA *rps16* intron sequences. *Am. J. Bot.* 88, 2064-2073.

Lewis, G., Schrire, B., Machinder, B., and Lock, M. (2005). *Legumes of the World*. Kew: The Royal Botanic Gardens.

Moteetee, A.N., Boatwright, J.S., and Jaca, T.P. (2012). A review of *Rhynchosia* section Cyanospermum (Phaseoleae, Fabaceae) in South Africa. *S. Afr. J. Bot.* 81, 124-127.

Ohashi, H. (2003). A new circumscription of *Tadehagi* and a new genus *Akschindlium* (Leguminosae). *J. Jap. Bot.* 78, 269-294.

Ohashi, H., Polhill, R.M., and Schubert, B.G. (1981). “Desmodieae,” in *Advances in Legume Systematics, Part 1.* eds. R.M. Polhill, and P.H. Raven (Kew: Royal Botanic Gardens), 292-300.

Ohashi, H. Nemoto, T., and Wu, T.L. (1996). The taxonomic position of *Trifidacanthus* (Leguminosae). *J. Jap. Bot.* 74, 84-95.

Ridder-Numan, J.W.A., and van der Ham, R.W.J.M. (1997). Pollen morphology of *Butea*, *Kunstleria*, *Meizotropis* and *Spatholobus* (Leguminosae, Papilionoideae), with notes on their position in the tribes Millettieae and Phaseoleae. *Rev. Palaeobot. Palyno.* 96, 255-280.

Sprent, J.I. (2007). Evolving ideas of legume evolution and diversity: a taxonomic perspective on the occurrence of nodulation. *New Phytol.* 174, 11-25.

Taberlet, P., Gielly, L., Pautou, G., and Bouvet, J. (1991). Universal primers for amplification of three non-coding regions of chloroplast DNA. *Plant Mol. Biol.* 17, 1105-1109.

Tiffney, B.H., and Mazer, S.J. (1995). Angiosperm growth habit dispersal and diversification reconsidered. *Evol. Ecol*. 9, 93-117.

Verdcourt, B. (1970). Studies in the Leguminosae-Papilionoideae for the Flora of Tropical East Africa. III. *Kew Bull.* 24, 379-447.

Verdú, M. (2002). Age at maturity and diversification in woody angiosperms. *Evolution* 56, 1352-1361.

Wojciechowski, M.F., Lavin, M., and Sanderson, M.J. (2004). A phylogeny of legumes (Leguminosae) based on analysis of the plastid matK gene resolves many well-supported subclades within the family. *Am. J. Bot.* 91, 1846-1862.

White, F. (1983). The vegetation of Africa: a descriptive memoir to accompany the UNESCO/AETFAT/UNSC vegetation map of Africa. Paris: UNESCO.

**Figure S1**
